# Training echo state networks for rotation-invariant bone marrow cell classification

**DOI:** 10.1007/s00521-016-2609-9

**Published:** 2016-09-21

**Authors:** Philipp Kainz, Harald Burgsteiner, Martin Asslaber, Helmut Ahammer

**Affiliations:** 1 Center for Physiological Medicine, Institute of Biophysics, Medical University of Graz, Graz, Austria; 2 Institute of Neuroinformatics, University of Zurich and ETH Zurich, Zurich, Switzerland; 3 Institute for eHealth, Graz University of Applied Sciences, Graz, Austria; 4 Institute of Pathology, Medical University of Graz, Graz, Austria

**Keywords:** Computer-assisted pathology, Histopathological image analysis, Bone marrow cell classification, Echo state networks, Reservoir computing

## Abstract

The main principle of diagnostic pathology is the reliable interpretation of individual cells in context of the tissue architecture. Especially a confident examination of bone marrow specimen is dependent on a valid classification of myeloid cells. In this work, we propose a novel rotation-invariant learning scheme for multi-class echo state networks (ESNs), which achieves very high performance in automated bone marrow cell classification. Based on representing static images as temporal sequence of rotations, we show how ESNs robustly recognize cells of arbitrary rotations by taking advantage of their short-term memory capacity. The performance of our approach is compared to a classification random forest that learns rotation-invariance in a conventional way by exhaustively training on multiple rotations of individual samples. The methods were evaluated on a human bone marrow image database consisting of granulopoietic and erythropoietic cells in different maturation stages. Our ESN approach to cell classification does not rely on segmentation of cells or manual feature extraction and can therefore directly be applied to image data.

## Introduction

The initial step of diagnostic work in histopathology is the assessment of cellularity in context of tissue architecture. Especially the diagnosis of bone marrow specimen requires a valid interpretation of different cell types with respect to their local distribution. Cell types of hematopoiesis, the process of blood stem cell maturation, are categorized into granulopoiesis, erythropoiesis, and megakaryopoiesis, which refer to maturation of white blood cells (WBC), red blood cells (RBC), and megakaryocytes, respectively [2].

**Fig. 1:**
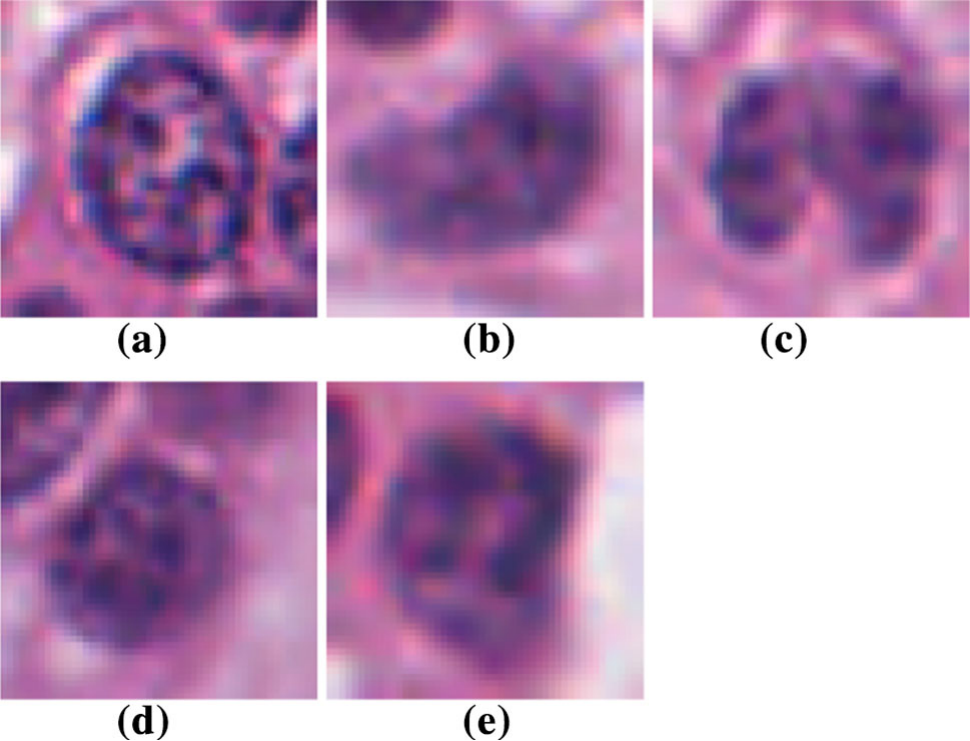
Samples of hematopoietic cell nuclei in the human bone marrow at $40\times$ magnification and stained with Hematoxylin–Eosin (H&E). Subsequent maturation stages of a granulopoietic cell: **a** myelocyte, **b** metamyelocyte, and **c** band cell. Especially in early stages, where the cells are not fully differentiated, different cell lineages share morphological characteristics: **d** myelocytes and **e** orthochromatic normoblasts (erythropoietic cells)

In healthy individuals, hematopoiesis mainly occurs in bone marrow, whereas extramedullary hematopoiesis is observed during fetal development, or may indicate pathological alterations [28]. In bone marrow specimen, several thousands of cells of multiple classes in different maturation levels have to be interpreted by the hematopathologist, and the class distributions need to be reported. This qualitative and semi-quantitative classification is usually performed on Hematoxylin and Eosin (H&E) stained tissue sections. The correct classification, based on cell morphology and spatial cell distribution, heavily dependents on the observer’s experience, since in hematopoiesis disparities between subsequent development stages are frequently indistinct and even equal maturation levels of different myeloid progenitor cells share morphological characteristics, cf. Fig. 1.

As a consequence, both inter- and intra-observer variability can be considerable, affecting the accurate diagnosis of reactive or even premalignant, and early malignant changes. Thus, automated image recognition systems exhibiting low variance, high classification accuracy, and predictable error are highly desirable for repeatable quantitative diagnostics [16]. Since virtual microscopy using whole slide images scanned at high magnification is emerging to a standard in pathology departments [1], computer-aided pathology using automated image analysis systems can easily be implemented in the routine diagnostic process.

### Related work

Over the recent years, a remarkable amount of research has been conducted on blood cell counting, segmentation, and classification in histopathological images for various applications. Motivated by the aggressiveness of blood cancer and the requirement for early diagnosis, most works were related to leukemia research, in particular identifying different types of leukemia by classifying WBC from histopathology images of peripheral blood smear [5, 17, 27, 36, 39, 40, 42–44, 48, 52] or bone marrow, obtained by aspiration [12, 35, 38, 41, 46, 49–51, 59, 60] and trephine biopsy [3]. Particularly, some work focused on classification of WBC in healthy tissue [5, 20, 39], while others dealt with detecting pathological alterations from morphological characteristics of cells [12, 41, 43, 59, 60].

Notably, a vast majority follows a conventional pattern recognition approach and used distinct steps for cell detection, segmentation, extraction of rotation and translation invariant features, and classification. Some studies mainly addressed detection and segmentation and relied on standard image processing techniques such as Hough space analysis [52], watershed transform, Gabor filters and adaptive thresholding [20], or intensity clustering [59, 60]. Others pursued supervised learning-based cell detection approaches using feed-forward neural networks (FF-NN) [45], fuzzy cellular neural networks [44], and random forests (RFs) [26]. Several approaches used statistical pattern recognition and classification techniques [22] such as support vector machines (SVM) [9, 17, 35, 46], FF-NN [17, 19, 27, 31, 36, 49–51], and Bayesian classifiers [6, 42, 43] to learn feature vectors representing individual cell objects. Decision tree-based methods such as regression trees [3], hierarchical trees using genetic algorithms for node optimization [56], or RFs [7, 12, 41] were used as well as *k*-nearest neighbor [37, 38, 41, 43], or heterogeneous classifier ensembles [12, 37]. Employing hierarchical models has been shown to be more powerful than using single-stage classifiers [46, 48].

Features related to shape and texture of cell nuclei were most frequently used and seemed to provide more discriminative power than statistical features from intensity histograms. This is reasonable, since—despite proper histopathological staining protocols—nuclei of different cell classes share very similar intensity patterns after staining [40], cf. Fig. 1. However, the choice and importance of features depend on the application. For instance, it was shown that features computed from cytoplasm can even be omitted for WBC classification and that the problem can be downscaled to using features from cell nuclei only [50]. In the context of another application, using features from both nuclei and cytoplasm resulted in higher classification performance [13]. Recent work of Reta et al. [41] on bone marrow cells concluded that features extracted from nuclei and cytoplasm separately are more discriminative than features from entire cells. In previous studies, the total number of features varied from a small set of four to over 190, comprising object-level features as well as global image features such as wavelet coefficients [36]. Nevertheless, handcrafting features from images require prior knowledge and experience, they are not easily transferable to other problems and may as well remove significant information, or introduce non-discriminative information. Thus, authors of previous papers frequently extracted feature candidates and applied automatic feature selection procedures to extract the most significant subset and hence compress the available information to achieve a better generalization performance [17, 37, 39, 42]. It has been shown that this strategy generally improved the classification results compared to using all available features [35, 42, 46] on specific problems. On the other hand, working directly on image intensity data provides a directly observable object representation that is not influenced by errors of preceding segmentation steps that are frequently inevitably to extract object-level features. Nevertheless, only a minority of previous work focused on learning a classifier from raw cell images [17, 44, 58], but reported promising results.

Very little work has been reported on quantitative analysis of bone marrow trephine biopsy images [3], or quantification of blood cell maturation [20]. Tissue micro-architecture is usually well preserved after histological preparation in bone marrow trephine biopsy samples. At the proper magnification, and using suitable histological staining protocols, this enables to inspect the morphological differences among subsequent maturation stages, but also introduces and emphasizes background structures irrelevant to cell classification. The most common stains used for tissue specimen were May–Grünwald–Giemsa (MGG), H&E (bone marrow), and Wright (peripheral blood), since morphological characteristics of objects of interest can be well represented. Feature-based discrimination of cells is usually less complicated in smear images depicting differentiated cells than in trephine biopsies: segmentation methods can more easily be applied to the cell objects without getting distracted by heterogeneous background. Despite the efforts of previous work, several issues have not yet been addressed, and the quantification of blood cell maturation in the bone marrow has not been sufficiently studied yet.

### Goals and organization of this paper

In this paper, we propose an alternative approach to bone marrow cell classification based on the direct application of a recurrent neural network (RNN) to images of H&E-stained images of bone marrow trephine biopsy. Under the conceptual framework of *reservoir computing*, two related effective training methods for an RNN have been developed independently: echo state networks (ESN) [21] and liquid state machines (LSM) [34]. Both approaches use a randomly and recurrently connected pool of hidden units and learn to classify the observed temporal activities by adapting the readout weights only. While LSMs are considered as a biologically more realistic model, applying ESN is usually easier due to a reduced number of hyper-parameters. Face recognition using a combination of ESN and FF-NN has been presented by Woodward and Ikegami [55], where the ESN extracts features, and inference is performed by the FF-NN. However, their approach did not yet consider any rotational invariant aspect.

**Fig. 2:**
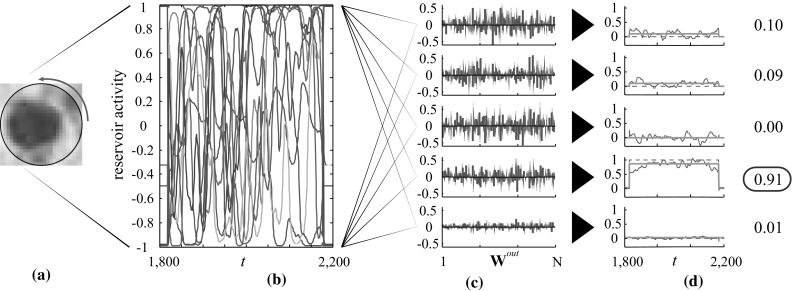
Proposed rotation-invariant multi-class ESN training scheme. Counterclockwise rotations of a cell patch **a** cause reservoir activity (i.e., feature computation) over time **b**. **c** For each class, a set of readout weights is learned. **d** The readout unit with the highest mean output (*green curve*) over the image presentation time finally determines the class. The *blue dashed curve* is the binary target function, which is set to one for the correct class and zero everywhere else. The *red curve* is the actual network output (color figure online)

The main motivation for this work is based on the fact that cells can appear under varying in-plane rotations. In a conventional rotation-invariant supervised learning setting, one could train exhaustively on samples representing independent rotations of the cell without taking into account the relations among consecutive rotations. Motivated by how RNN can capture appearance information in temporal features, we propose a rotation-invariant learning scheme for cell classification using pure ESN, as can be seen in Fig. 2. This work shows that it is possible to train an ESN with standard ridge regression directly on raw image data in a way that its classification accuracy is completely independent from the rotation of the cell. While previous work heavily relied on explicit feature extraction from segmented cells, nuclei. or cytoplasm, our approach does not include such steps and can be applied on cell image patches directly. We can omit a dedicated segmentation step of cell nuclei, and cytoplasm, which in fact is not always possible in our cell samples, cf. Fig. 1. Based on results from our earlier work [24, 25], where a similar, but only binary cell recognition problem was considered, we also explored the extension of this approach to a multi-class problem [23]. In addition to [23], this contribution is extended by providing a direct comparison with an RF [7] image classifier that was trained the conventional way to achieve rotation-invariance. An RF is an ensemble of de-correlated, binary decision trees that are individually trained and produce a consolidated prediction. In machine learning and computer vision, forests are well known for their efficiency and good generalization ability without the tendency to overfit the data [11, 18]. Further, they perform well on unbalanced and small datasets, have become a key ingredient in patch-based medical image analysis [10, 14, 30, 57], and have recently shown state-of-the-art performance on similar bone marrow data [26]. Further, this work discusses strengths and weaknesses of both approaches.

In the subsequent sections, we elaborate on the novel rotation-invariant training scheme for ESN, provide the outline of our experimental setups, and present the results. The findings are discussed and an outlook to future work is given in the final section.

## Methods

**Fig. 3:**
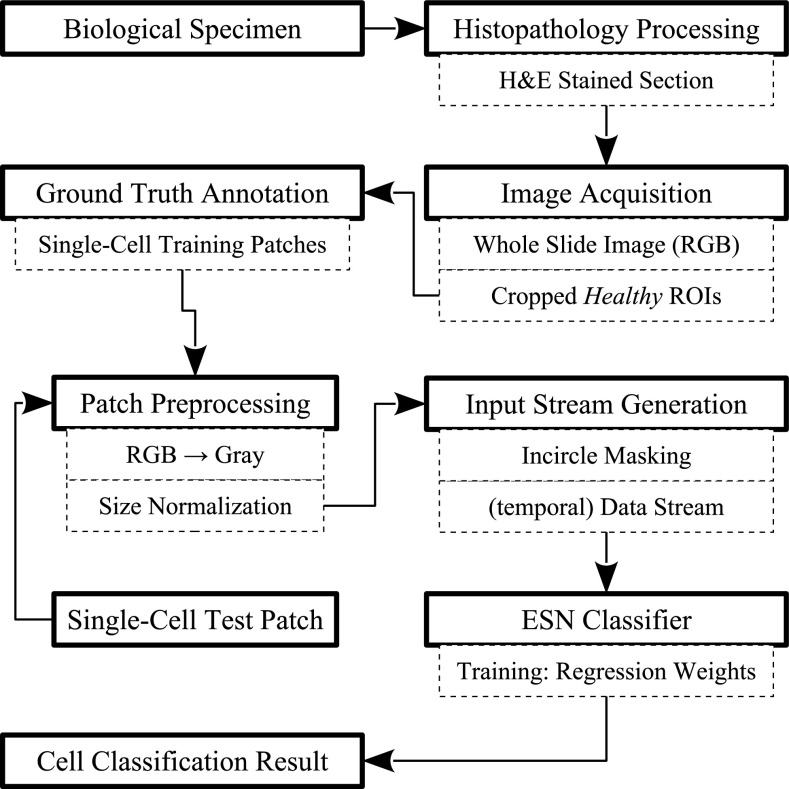
Overview of the cell recognition pipeline. Biological tissue specimen is prepared in the histopathology laboratory according to standard protocols for H&E staining. The sections are digitized to RGB whole slide images, and regions of interest (ROIs) containing healthy tissue are cropped. Typical bone marrow cells of four classes are labeled as ground truth by an expert pathologist. For this work, single-cell RGB patches are manually extracted as part of the ground truth labeling, converted to gray-scale, and resized. A temporal input data stream is generated by rotating the incircle-masked static images, which is then fed into the ESN classifier, cf. Fig. 2. During training, ridge regression determines a set of weights that can be used to predict the class label of a test patch using a threshold-based inference scheme

We propose an ESN approach for rotation-invariant blood cell maturity recognition in the human bone marrow. An overview of our classification scheme starting with the biological sample is illustrated in Fig. 3. Our supervised learning system is trained with image patches, which are first labeled by an experienced pathologist as one of multiple foreground classes, or background. We omit automatic cell detection in this work and focus on classifying manually cropped image patches. Nevertheless, we discuss a potential cell localization method in Sect. 5 that will eventually be required when considering this approach in an integrated system.

### Multi-class echo state networks

Echo state networks (ESNs) are a way to train RNNs for temporal prediction tasks [21]. Many different ESN architectures have been proposed [33], but in this work we focus on the classical architecture [21]. The reservoir, a randomly connected RNN composed of *N* units, models short-term memory and nonlinear input expansion. Reservoirs in ESNs have to ensure the ‘echo state’ property to be an universal function approximator. Hence, the recurrent reservoir weights ${\mathbf {W}}\in {\mathbb {R}}^{N\times N}$ must be scaled, such that the spectral radius $\rho ({\mathbf {W}})<1$ [21]. In practice, the spectral radius is a global control parameter that defines how fast the reservoir activity vanishes [32]. Hence, a larger spectral radius results in slower decay and longer interaction of reservoir activity.

**Fig. 4:**
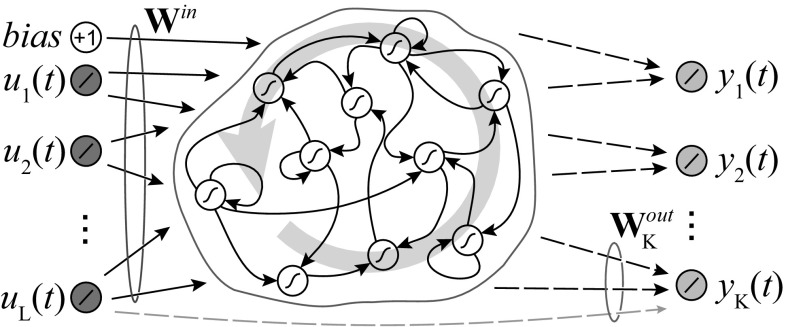
Multi-class ESN architecture. At each time step *t*, *L* linear input units (*green*) feed input ${\mathbf {u}}(t)$ into the reservoir via input weights ${\mathbf {W}}^\mathrm{in}$. Each of the *K* linear readout units (*blue*) corresponds to a specific class. The reservoir consists of *N* (internal) units with hyperbolic tangent (tan*h*) activation function. Readout-to-reservoir feedback connections are omitted in our architecture. The input layer is fully connected to each readout unit, symbolically illustrated for one unit by the *gray dashed arrow* at the *bottom*. This provides contextual information on the original input in parallel to the temporal features. After learning readout weights ${\mathbf {W}}^\mathrm{out}_c$, the output $y_c(t)$ is determined for the readout units (color figure online)

Figure 4 shows the architecture of a multi-class ESN. The *L*-dimensional input at particular point in time *t*, given by ${\mathbf {u}}(t)=[u_1(t), u_2(t),\ldots ,u_L(t)]^{\mathrm {T}}$, and a bias unit is connected to the reservoir units via the input weights ${\mathbf {W}}^\mathrm{in}\in {\mathbb {R}}^{N\times (1+L)}$. When an input is presented to the input layer, it causes nonlinear activity in the reservoir. This activity represents the (temporal) features, the recurrent reservoir units compute from the input stimulus at each observable time step. The weights ${\mathbf {W}}^\mathrm{in}$ and ${\mathbf {W}}$ may be sparse and remain fixed after random initialization and meeting task-specific scaling criteria [53].

A binary classification task can already be performed by a single readout unit. Given a training set of one positive class and one negative class, the unit is trained to recognize samples belonging to the positive class and ignore the negative samples. This simple scheme can easily be extended to solve multi-class problems: each class $C_c, c\in \lbrace 1,\ldots ,K\rbrace$ is represented by a single readout unit, which is trained in the *one-versus-all* scheme.

When stimulating the reservoir, the state update equation at time step $t+1$ is given as

1 $${\mathbf {x}}(t+1) = (1-\alpha ) {\mathbf {x}}(t) + \alpha {\tan }h({\mathbf {W}}^\mathrm{in}[1;{\mathbf {u}}(t)] + {\mathbf {Wx}}(t)),$$

where ${\mathbf {x}}(t)$ denotes the state vector at the previous time step *t*. The leaking rate $\alpha$ defines the short-term memory capacity, i.e., how strong the reservoir activity at time step *t* influences activity at $t+1$. The ESN can be set to a generative mode, where the input ${\mathbf {u}}(t)$ is switched off. We use the term *generative mode* here to avoid confusion with the very similar *pattern generator* principle [32]. The difference is that we do not use output-to-reservoir feedback or apply the output at *t* as input at $t+1$, but compute the state updates purely from the remaining activity within the reservoir using

2 $${\mathbf {x}}(t+1) = (1-\alpha ) {\mathbf {x}}(t) + \alpha {\tan }h({\mathbf {Wx}}(t)).$$

During a recording time $\varPsi$, input and state vectors are concatenated in a large state matrix ${\mathbf {X}}\in {\mathbb {R}}^{(1+L+N)\times \varPsi }$. Using a single reservoir facilitates automatically capturing the activities caused by multiple classes. The individual readout weights ${\mathbf {W}}^\mathrm{out}_c\in {\mathbb {R}}^{(1+L+N)}$ for $C_c$ are learned via ridge regression with Tikhonov regularization

3 $${\mathbf {W}}^\mathrm{out}_c = {\mathbf {Y}}^\mathrm{target}_{c}{\mathbf {X}}^{\mathrm {T}} ({\mathbf {X}}^{\mathrm {T}}{\mathbf {X}}+\beta {\mathbf {I}})^{-1},$$

where ${\mathbf {Y}}^\mathrm{target}_{c}\in {\mathbb {R}}^{\varPsi }$ denotes the desired target function of class $C_c$, ${\mathbf {X}}^{\mathrm {T}}$ the transpose of the state matrix, and ${\mathbf {I}}$ the identity matrix. The Tikhonov regularization coefficient is fixed to $\beta =10^{-2}$.

A piece-wise constant target function is regressed for each class $C_c$ at a recorded time step *t*, which is given by

4 $$y^{\mathrm{target}}_{c}(t) = {\left\{ \begin{array}{ll} 1 &{} \text {if }\, {\mathbf {u}}(t) \in C_c \\ 0 &{} \text {otherwise} \end{array}\right. }.$$

Each readout unit produces an output ${\mathbf {Y}}_c=\mathbf {W}^\mathrm{out}_c {\mathbf {X}}$, and a score over a predefined inference period $\varOmega$, computed as the mean output (score)

5 $$\bar{y}_c=\frac{1}{\vert {\varOmega }\vert }\sum _{t=-{\varOmega }/2}^{{\varOmega }/2}y_c(t).$$

The unit with the highest mean score subsequently determines the winner class

6 $$c^*=\mathop {\mathrm{arg}\,\mathrm{max}}\limits _{c} \lbrace \bar{y}_c\rbrace.$$

### Rotation-invariant cell classification

**Fig. 5:**
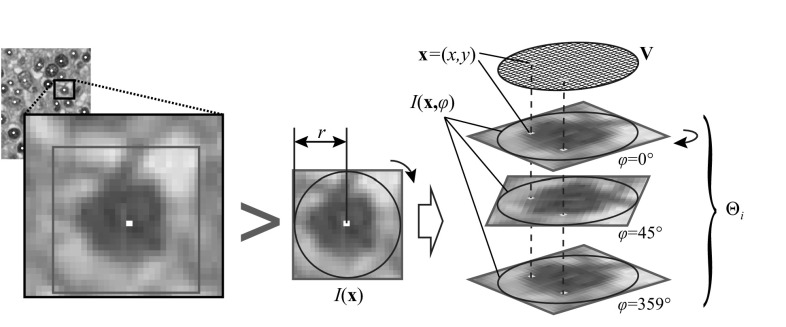
Illustration of the input stream generation for the rotation-invariant learning scheme. A patch containing a single cell is extracted from a virtual slide. It is then normalized to a predefined size $2r\times 2r$ to fit a receptive field ${\mathbf {V}}$. A static image patch $I({\mathbf {x}})$ is transformed into a stream $\mathrm {\Theta }_i$ by concatenating subsequent rotations $I({\mathbf {x}},\varphi )$. For each rotation, ${\mathbf {V}}$ forwards the pixel intensity within the incircle of $I({\mathbf {x}},\varphi )$ into the reservoir

Given an image patch $I({\mathbf {x}})$ centered on a single cell at an image location ${\mathbf {x}}=(x,y)$, we need to transform it into time-dependent input for the ESN classifier. In order to generate temporal input from $I({\mathbf {x}})$, we take advantage of the fact that cells can occur in arbitrary rotations within tissue. Figure 5 illustrates the generation of a temporal input stream $\mathrm {\Theta }_i$ for a cell by concatenating subsequent rotations of the patch $I({\mathbf {x}},\varphi )$. While rotating by an angle $\varphi$, we ignore the patch corners and just consider the pixels within the incircle radius *r*. For this purpose, a receptive field ${\mathbf {V}}$ of radius *r* is defined as the input layer and forwards the pixel intensities into the reservoir. All patches are required to be normalized to a fixed size of $2r\times 2r$ beforehand.

Rotation-invariance is achieved by letting the reservoir to generate features for each $I({\mathbf {x}},\varphi )$, $\varphi =0^\circ ,\ldots ,{359}^\circ$, starting at an arbitrary angle $\varphi _0$ that relates to a cell’s arbitrary orientation in a slide. These reservoir states are harvested by evaluating Eq. (1), and the target function is approximated at each recorded time step. After the network saw all $I({\mathbf {x}},\varphi )$, the final class is determined with Eq. (6).

The generative mode of ESNs also enables skipping $\Delta \varphi$ rotations after receiving external input $I({\mathbf {x}},\varphi )$. Due to the memory and decaying reservoir activity we are still able to obtain discriminative features using Eq. (2), even without external input driving the reservoir. The reservoir activity usually approaches a resting state without external input and thus a properly selected $\rho ({\mathbf {W}})$ ensures that there is enough activity left before the next input $I({\mathbf {x}},\varphi +\Delta \varphi )$ is presented.

In general, two kinds of memory can be observed in an ESN. Firstly, memory that can be controlled by the leaking rate parameter $\alpha$, we name ‘state’ memory in this context. Secondly, the ‘immutable’ memory, which is inherently modeled by the recurrent weights ${\mathbf {W}}$ in the reservoir. The state memory models the influence of previously computed reservoir states on the current state and therefore controls smoothing of the temporal features. If $\alpha \ll 1$, the internal states caused by previous rotations may significantly influence subsequent ones and may cause over-smoothed states. On the other hand, if $\alpha =1$, the state memory is turned off and features for each observed rotation $I({\mathbf {x}},\varphi )$ are less influenced by previous states. However, setting $\alpha =1$ does not entirely turn off the memory capacity of the reservoir, since the recurrent connections defined by the internal weights ${\mathbf {W}}$ are not affected. To find a suitable amount of state smoothing, $\alpha$ needs to be chosen accordingly.

## Experimental setup

### Bone marrow cell dataset

We challenge our approach on a non-neoplastic human bone marrow cell dataset composed of three consecutive maturation stages in granulopoiesis as well as one class from erythropoiesis, as can be seen in Table 1. Myelocytes, metamyelocytes, and band cells are three consecutive maturation stages of WBC in the bone marrow and are characterized by a high intra-class variability and a small inter-class distance. Biological samples were taken from the human iliac crest by trephine biopsy, embedded in acrylate, cut into slices of $\approx 2\,\upmu \hbox {m}$, and stained with H&E. Cell patches were extracted from virtual slides of two patients (digitized at $40\times$ magnification using an Aperio whole slide scanner) and labeled by an expert pathologist. All cells appeared at the same object scale. Considering the problem of hematopoietic cell classification without megakaryocytes, the object scale within a class does not vary by more than a factor of approximately $\pm 0.2$, which still can be compensated by our approach. The total number of original patches were extended by a factor of six using nonlinear warping transformations (circular distortion by $\pm {35}^\circ$) as well as horizontal flipping, resulting in 744 foreground patches of four classes. Despite this data augmentation strategy, we ensured that both transformed and original images were unique, and that rotation of the extended dataset did not introduce any duplicates. In addition, we used 200 randomly sampled background patches from the same virtual slides as control class. With respect to all positive classes (i.e., foreground classes), the control class served as negative class. Hence, samples of this class did not contain any centered cells. It was used to verify the capability of a classifier to discriminate among different positive classes, as well as between all positive classes and the negative class. All patches were converted to gray-scale by averaging the color channels (RGB mean). At $40\times$ magnification, the average single-cell patch size in our dataset was $33\times 33$ pixels. Hence, we could normalize the patches to a fixed size of $20\times 20$ pixels using bilinear interpolation without losing significant appearance information or introducing artifacts. The receptive field of the ESN was connected to all incircle ($r=10$) pixels of that patch, resulting in $L=332$ network inputs. In total, the dataset comprised $n=944$ patches, of which $66\,\%$ ($n=623$) were assigned for training and hyper-parameter optimization, and $34\,\%$ ($n=321$) were held-out for testing.

**Table 1:**
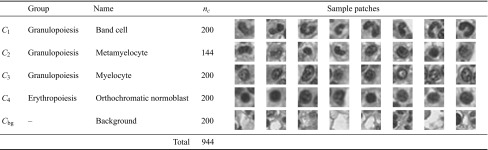
The non-neoplastic bone marrow dataset used for method evaluations consists of a total of $n=944$ patches

### Echo state network

In order to avoid learning the sequence of cell classes rather than the appearance of each cell, we introduced periods with zero-input of random length between two consecutive cell image patches. They were furthermore required to let the reservoir ‘forget’ about the previous image and learn each instance separately. Depending on the spectral radius $\rho ({\mathbf {W}})$, the network approximately required 20–50 time steps to reach the resting state after the last input stimulus has been presented. Therefore, we randomly sampled the zero-input length in the range [50, 100]. Please note that this is a different concept than setting the network to the generative mode after presenting an image input, because we did not apply another input stimulus as long as it did not completely reach the resting state.

Starting with ESN hyper-parameters for a binary classification task [24] determined by simulated annealing [4], we continued with manual fine-tuning. Considering the influence of the hyper-parameters to achieve higher performance [32], they were successively optimized for our cell recognition task using tenfold cross-validation (CV) on the training set ($n=623$). Dense connectivity was used in the input weights ${\mathbf {W}}^\mathrm{in}$, while only $30\,\%$ of the reservoir connections ${\mathbf {W}}$ were nonzero. The normalized pixel intensity was bounded within [0, 1], so we shifted and scaled it to $[-1,1]$ to avoid using only the linear part of the tan*h* activation function of the reservoir units. For a presented input $I({\mathbf {x}})$, all readout units showed rather high activity in the first and last few recording steps. Since these reservoir activities did not contribute to the actual classification, we bounded the inference window ${\varOmega }$ to start $5\,\%$ after the first and to end $5\,\%$ before the last sample.

### Random forest

The performance of the ESN was compared to a classification random forest [7]. We trained a standard RF to solve the same five-class classification problem that was previously defined for the ESN. In analogy to the ESN setup, the input data for the RF were the gray-value single-cell patches. Training a decision tree in a forest is based on the principle that the dataset arriving at an internal node *j* gets split into a left and right subset based on randomly selected criteria. The optimization problem at each node is given by maximizing an objective function to find the best split decision. The quality of the split decision was evaluated using the Gini index [8] as the objective function that measures the ‘class purity’ in each of the subsets. Suitable hyper-parameters such as number of random node tests, number of samples to test a split, and split functions were evaluated in tenfold cross-validation experiments. We used a simple split function that randomly selected two locations in the image patch and compared the intensity difference to a randomly selected threshold. A common rule uses $\left\lfloor \sqrt{p}\right\rfloor$ random node tests for each split [18], where *p* is the number of features per sample. For $20\times 20$ pixels images, each pixel being a feature, this results in only 20 node tests ($p=400$). However, we found that increasing the number of split function tests per node to 100 and comparing each one to 20 random thresholds (in 2000 tests per node) resulted in better performance. Each split decision was evaluated on 200 samples that were randomly selected from the data available at a node *j*. A terminal (leaf) node was constructed when either the maximum tree depth was reached or the size of the dataset arriving at node *j* was smaller than a predefined number of 100 samples. Leaf nodes store the class histogram of the data. Bagging did not result in higher performance in the forest training. Hence, each tree was learned all available training data samples. Once the trees were constructed, we could propagate an image patch $I({\mathbf {x}})$ through the forest and the final ensemble output was produced by averaging the decisions of the individual trees, producing a probability distribution over the class labels.

### Classification performance metrics

Both ESN and RF classification performance were assessed quantitatively. The overall performance is reported as mean accuracy (ACC) weighted by the class distribution

7 $${\hbox {ACC}}=\frac{1}{n} \sum _c{n_c\,{\hbox {TP}}_c},$$

with *n* as the total number of samples and $n_c$ as the number of samples in class $C_c$. ${\hbox {TP}}_c$ denotes true positive, ${\hbox {FP}}_c$ false positive, ${\hbox {TN}}_c$ true negative, and ${\hbox {FN}}_c$ false negative predictions for class $C_c$. In order to observe the performance at the class level, more detailed measures than the overall accuracy were required. Class-wise performance is reported as precision (${\hbox {PRC}}_c$), recall (${\hbox {REC}}_c$), specificity (${\hbox {SPC}}_c$), and F1-score ($\mathrm{F1}_c$):

8 $${\hbox {PRC}}_c=\frac{{\hbox {TP}}_c}{{\hbox {TP}}_c+{\hbox {FP}}_c},$$

9 $${\hbox {REC}}_c= \frac{{\hbox {TP}}_c}{{\hbox {TP}}_c+{\hbox {FN}}_c},$$

10 $${\hbox {SPC}}_c= \frac{{\hbox {TN}}_c}{{\hbox {TN}}_c+{\hbox {FP}}_c},$$

11 $$\mathrm{F1}_c=\frac{2\cdot {\hbox {PRC}}_c\cdot{\hbox {REC}}_c}{ {\hbox {PRC}}_c + {\hbox {REC}}_c}.$$

Actual values of performance measures are reported as mean and standard deviation (SD).

### Experiment definitions

We were interested in the performance of the proposed rotation-invariant approach in terms of overall and class-wise accuracy under different conditions. Therefore, we defined the following four experimental settings, where for each individual cell image 360 reservoir states were collected. We used bilinear interpolation when rotating the images to reduce artifacts caused by aliasing.

*Experiment 1*: In the first experiment we were interested in the general applicability of the proposed approach to multi-class cell classification. The ESN was trained on a sequence of all possible (integer) rotation angles $\varphi = 0^\circ ,\ldots ,{359}^\circ$ of an individual image patch. Hence, no generative mode was used ($\Delta \varphi =0$).
*Experiment 2*: In the second experiment the generative mode of the ESN in the context of image classification was evaluated. In particular, we focused on whether or not meaningful features could be computed when we inserted a predefined stimulus-free period (‘zero-input’) between showing single rotations of the image. Instead of having 360 individual inputs from an image, we skipped five subsequent rotation angles ($\Delta \varphi =5$) and recorded the decaying reservoir activity. For instance, the input stimulus sequence for the first 12 time steps included three actual image inputs at the rotation angles ${0}^\circ$, ${6}^\circ$, and ${12}^\circ$, respectively: $[{\mathbf {u}}(t_0) = I({\mathbf {x}},0), \; {\mathbf {u}}(t_{1,\ldots ,5}) = 0, \; {\mathbf {u}}(t_6) = I({\mathbf {x}},6), \; {\mathbf {u}}(t_{7,\ldots ,11}) = 0, \; {\mathbf {u}}(t_{12})=I({\mathbf {x}},12), \; \ldots ]$.
*Experiment 3*: Here, we increased the duration of the generative mode and skipped 10 rotation angles ($\Delta \varphi =10$).
*Experiment 4*: To simulate a concrete real-world application, the classifier is learned from scratch on all available training data ($n=623$) and tested on the held-out test data ($n=321$). Further, we used $\Delta \varphi =5$ (see also experiment 2) to examine whether the ESN is able to deal with short periodical, but different input stimuli, and compared it to the RF trained the conventional way to achieve rotation-invariance. A classifier is considered to be invariant to rotations, if the very same object is always labeled with the same class label under arbitrary in-plane rotations. Hence we examined, whether both ESN and RF were able to recognize the very same cell again under a randomly selected, different rotation angle. The number of samples per class in the test set was $n_1=69$, $n_2=56$, $n_3=61$, $n_4=65$, and $n_\mathrm{bg}=70$, which also approximately reflected the distribution in the training set. We did not perform any additional data augmentation to balance the training set.

Using the settings from experiments 1–3, we examined the influence of the generative mode on the classification performance of the ESN in tenfold CV experiments on the training set. The size of the reservoir was varied ($N=\lbrace 200, 500, 1000, 2000, 3000, 4000\rbrace$) to assess the required memory capacity of the ESN for the tasks. For each *N*, the reported values correspond to the best results of the hyper-parameter fine-tuning, which was stopped once the performance reached saturation on our dataset. A similar approach was carried out for the RF, where we varied the number of individual trees and their depth along other hyper-parameters determined by the preceding search. The samples in the dataset were randomly shuffled at the beginning of the experiments. Despite that the main focus of this work was set on evaluating the rotation-invariant ESN classifier, we employed the previously described RF as baseline classifiers to validate the results.

## Results

### Model evaluations

#### Echo state network

The classification performance of the proposed rotation-invariant approach was evaluated in terms of weighted mean overall accuracy (ACC). Five independent tenfold CVs were run in experimental settings 1–3, cf. Sect. 3.5. In order to fix a suitable amount of short-term memory, prevent over-smoothing the state space, and account for proper reservoir decay, the following parameter tuples $(\Delta \varphi ,\rho ({\mathbf {W}}),\alpha )$ were used for the ESN experiments: (0, 0.6, 0.85), (5, 0.8, 0.85), and (10, 0.95, 0.85). We collected 360 reservoir states for each individual cell image patch, starting at rotation $\varphi _0=0$.

**Fig. 6:**
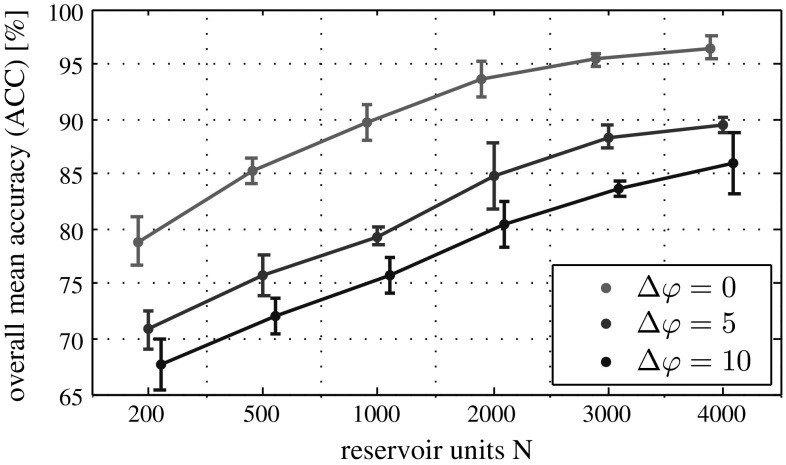
Performance evaluation of the ESN model. Mean weighted accuracy (ACC) over five independent tenfold CV experiments, *error bars* refer to three standard deviations (SD). The longer the ESN is in generative mode, the more reservoir units are required to generate sufficient features for classification via linear regression

Quantitative results of the CVs are reported in Table 2 and visualized in Fig. 6. Obtaining higher performance generally required larger reservoirs. For instance, to reach approximately $80\,\%$ accuracy, it took ten times more reservoir units to get similar performance in experiment 3 than in experiment 1: ($N=2000$, $\Delta \varphi = 10$) versus ($N=200$, $\Delta \varphi =0$), cf. Table 2.

**Table 2:** Results of the ESN model evaluations

Exp.	$\Delta \varphi$		Reservoir size *N*					
			200	500	1000	2000	3000	4000
1	0	ACC	78.85	85.32	89.76	93.63	95.37	96.43
		SD	(0.75)	(0.37)	(0.55)	(0.51)	(0.17)	(0.35)
2	5	ACC	70.82	75.85	79.37	84.73	88.39	89.49
		SD	(0.59)	(0.63)	(0.27)	(1.01)	(0.35)	(0.22)
3	10	ACC	67.72	72.08	75.82	80.42	83.64	85.96
		SD	(0.77)	(0.53)	(0.54)	(0.70)	(0.22)	(0.92)

Five independent tenfold cross-validation experiments were run on the training dataset. Values are reported in percent as mean weighted accuracy (ACC) and standard deviation (SD), see also Fig. 6

**Fig. 7:**
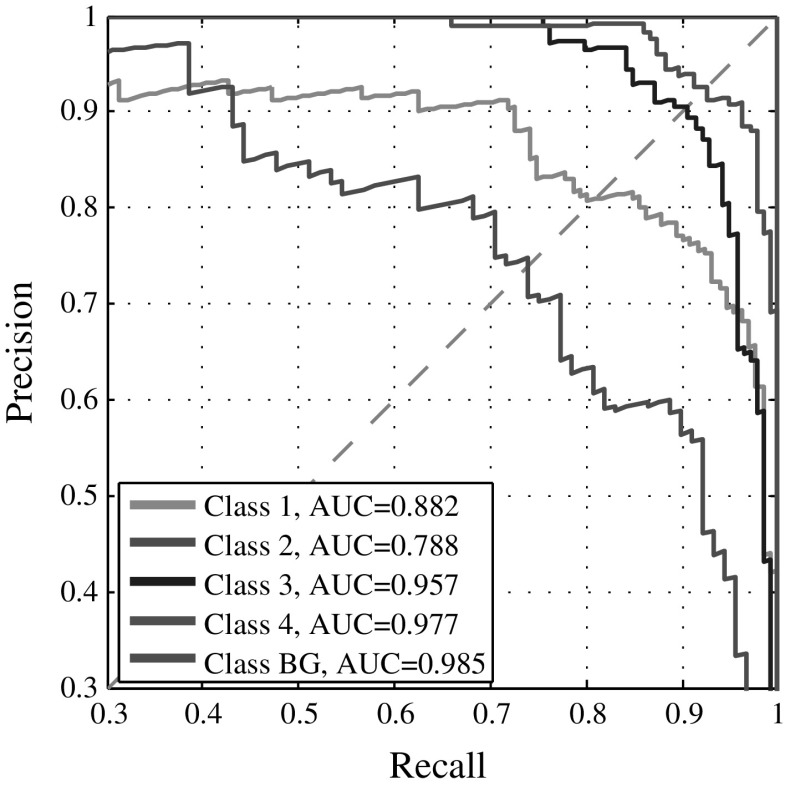
Precision–recall curve for one ESN cross-validation experiment with $N=1000$ and $\Delta \varphi =0$. The area under the curve (AUC) for $C_1$ and $C_2$ is lower than for the other classes. With larger reservoirs even these two classes could obtain higher performance

Discriminating subsequent maturation stages of granulopoietic cells (i.e., $C_1$, $C_2$, and $C_3$) was challenging for the ESN. Taking a closer look at a single CV run from experiment 1 with $N=1000$ and $\Delta \varphi =0$, we observed that this task required larger reservoirs to capture the subtle differences in the cells’ appearance. Considering the area under the curve (AUC) in Fig. 7, learning to recognize band cells ($C_1$) and metamyelocytes ($C_2$) in this setting seems to be harder than learning the other classes. The confusion among these classes may be caused by their indistinct class borders, because we did not observe this effect among $C_3$ and $C_4$. All samples of the background class $C_\mathrm{bg}$ could be recognized correctly.

#### Random forest

Similarly, the dataset for the baseline classifier (RF) experiments was augmented with the same number of rotations and rotation angles that were available as inputs to the ESN (i.e., $360/\mathrm {max}\lbrace \Delta \varphi ,1\rbrace$). In a grid search, 16 combinations of two main RF parameters were evaluated: the number of individual trees, i.e., *forest size* $\, =\lbrace 4,16,64,128\rbrace$, and the *maximum tree depth* $\, =\lbrace 2,4,8,12\rbrace$.

**Fig. 8:**
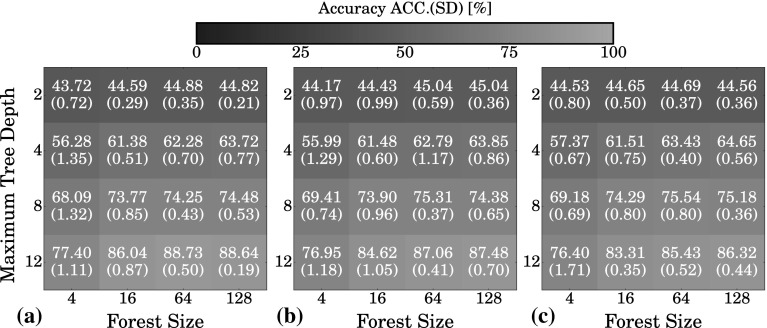
Results of the experiments to select the best RF model. In a grid search, 16 combinations of two main RF parameters *forest size*, i.e., the number of trees, and *maximum tree depth* were evaluated. Performance values for **a** $\Delta \varphi =0$, **b** $\Delta \varphi =5$ and **c** $\Delta \varphi =10$ are reported in percent as weighted mean accuracy (ACC) and standard deviation (SD, *in brackets*). Higher overall mean accuracy corresponds to *green color*, where low accuracy is represented by *blue color* (color figure online)

Like for the ESN, five independent tenfold CVs were run on the training dataset using the RF. Figure 8 illustrates the results. The classification accuracy could significantly be improved when larger and deeper forests were used. However, with respect to higher performance, the depth of the individual trees was more important than the total number of trees in the forest.

#### Classifier comparison

Compared to the ESN, the RF was less sensitive to omitted rotation angles. The difference between maximum and minimum mean accuracy among experiment 1–3 was in the range of 0.1–3.3 % for the RF (Fig. 8), and in the range of 10.5–14.0 % for the ESN. This may be explained by the fact that in the RF training, each rotation was considered as an individual sample, while the ESN received a continuous stream formed by the rotations of an image patch and omitting rotations causes unforeseen interruptions. These interruptions could only be compensated by larger reservoirs. Conversely, this also indicates that training a RF on each possible integer rotation ($\Delta \varphi =0$) is not necessary, and using much less training data already results in similarly high performance.

### Robustness for random starting angles $\varphi _0$

We have shown in experiment 1 that generalization of an ESN works well when it is trained on all 360 rotations of an image patch. Further, it is also capable of learning a classifier that works with periods of zero-input between two consecutive rotations (experiments 2–3). The CV experiments suggested that increasing the reservoir size also increases the classification performance on all classes, cf. Fig. 6. Using $\varphi _0=0$ and $\Delta \varphi =5$ as reference scheme, we examined whether the very same cells could be recognized equally well when the rotation started at a random $\varphi _0$. Therefore, the best ESN and RF models were selected from the CVs with respect to experiment 2 (ESN: $N=4000$, RF: *forest size* $\, = 128$, *maximum tree depth* $\, = 12$).

**Fig. 9:**
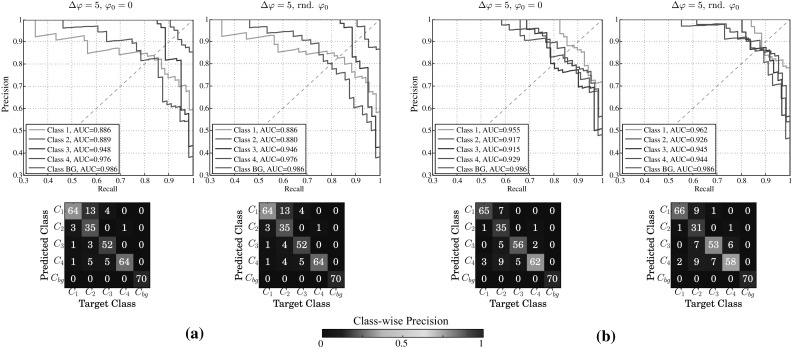
Classifier performance on the test set in experiment 4 ($n=321$, $\Delta \varphi =5$). The *top row* shows precision–recall curves and the area under the curve (AUC), the *bottom row* the confusion matrices. The *first column* in **a** illustrates the results for the ESN classifier trained on the original initial starting angles $\varphi _0=0$, while the *second column* refers to results obtained using a random $\varphi _0$. Similarly, in **b** the results are illustrated for the RF classifier. The *color bar* encodes the class-wise precision (i.e., the ratio of true positives per predicted class), colors toward *red* correspond to higher precision, i.e., less false positives. While the ESN frequently confused $C_1$ and $C_2$, the predominant misclassifications of the RF were among $C_3$ and $C_4$. However, the RF showed better results for $C_1$ and $C_2$ (color figure online)

**Fig. 10:**
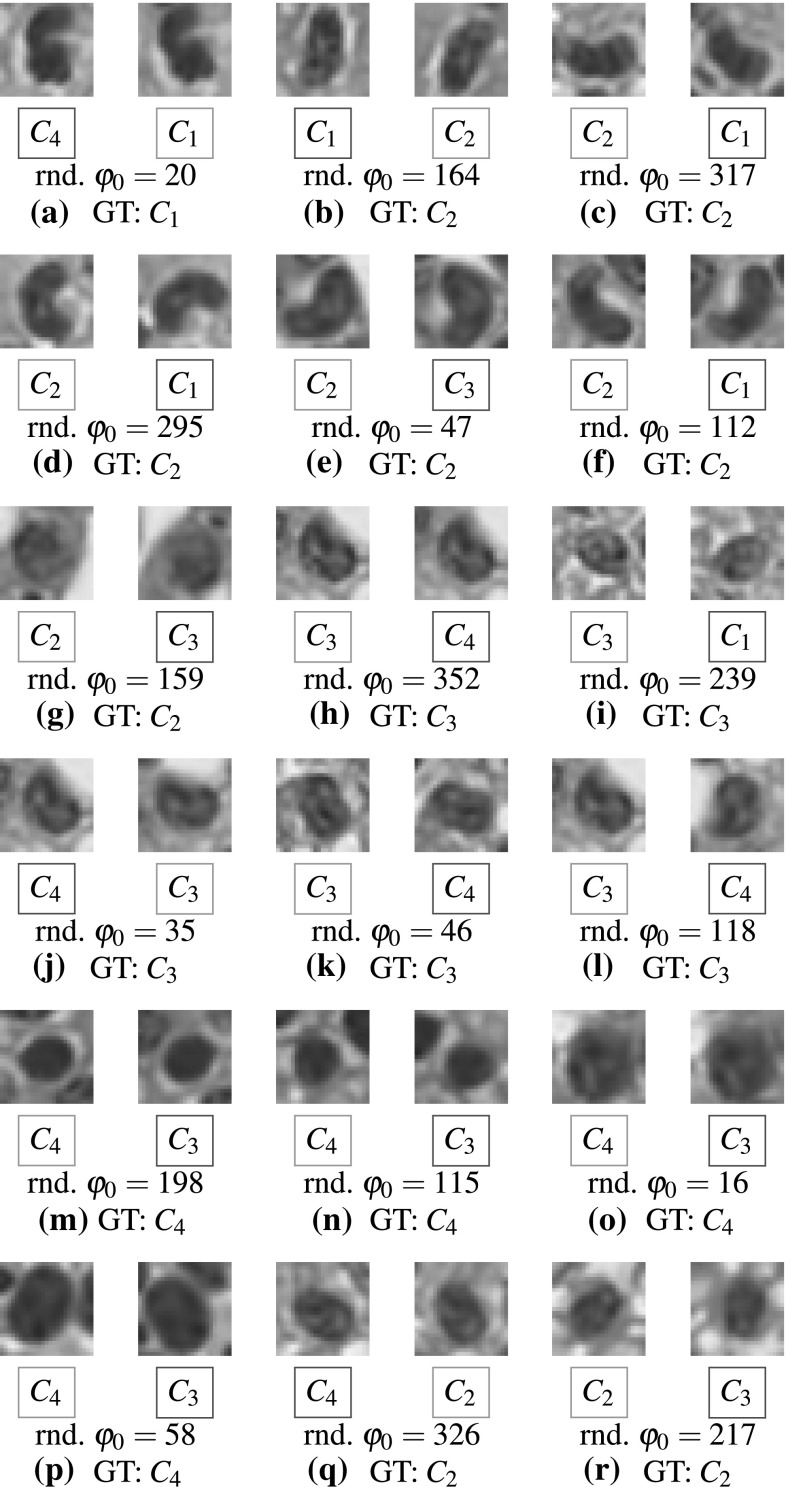
16 samples (4.98 %) classified differently by the RF classifier in experiment 4 (**a**–**p**), and the two samples (0.62 %) classified differently by the ESN (**q**, **r**). The *left image* shows the original cell patch at $\varphi _0=0$, the *right image* at a random starting angle (rnd. $\varphi _0$). Below these images the predicted classes are *enclosed in green boxes*, if they were recognized as true positives, or in *red boxes* otherwise. The used counterclockwise initial rotation angle and ground truth class (GT) are shown below the predictions (color figure online)

In Fig. 9 we report precision–recall curves, AUCs, and the confusion matrices on the fixed test set ($n=321$) for both classifiers. Generally, the results were of almost equal quality with respect to the weighted mean measures for a patch rotation starting at $\varphi _0=0$ and random $\varphi _0$. Considering precision, the ESN achieved 89.40 and 89.30 %, the RF 90.50 and 87.60 %, respectively. The recall of the ESN is constant 88.80 %, but marginally decreases for the RF from 89.70 to 86.60 %. While only two out of 321 cells (0.62 %, Fig. 10q, r) were predicted differently by the ESN, the RF predicted 16 cells (4.98 %, Fig. 10a–p) differently. Classification measures reported in Table 3 are more stable for the ESN than for the RF. More specifically, very stable precision and recall values indicate that the recognition accuracy does not necessarily depend on the initial rotation angle and that the proposed approach to train ESNs for image recognition works very well. The absolute value differences between $\varphi _0=0$ and random $\varphi _0$, denoted as $\vert \Delta \vert$, is close to zero for most of the measures. These results suggest that the ESN is able to robustly predict the same class label of a particular cell in 99.38 % of all test cases, even if $\varphi _0$ randomly falls within $\Delta \varphi$, where the network is in the generative mode. However, the baseline classifier (RF) achieved comparable performance in almost all measures, cf. Table 3.

**Table 3:** Class-wise performance of the ESN and RF on the fixed test dataset ($n=321$) in experiment 4

		PRC			REC			SPC			F1-score		
		$\varphi _0 = 0$	rnd. $\varphi _0$	$\vert \Delta \vert$	$\varphi _0 = 0$	rnd. $\varphi _0$	$\vert \Delta \vert$	$\varphi _0 = 0$	rnd. $\varphi _0$	$\vert \Delta \vert$	$\varphi _0 = 0$	rnd. $\varphi _0$	$\vert \Delta \vert$
$C_1$	ESN	0.791	0.791	**0.000**	0.928	0.928	**0.000**	0.934	0.934	**0.000**	0.853	0.853	**0.000**
$n_1=69$	RF	**0.903**	**0.868**	0.035	**0.942**	**0.957**	0.015	**0.972**	**0.960**	0.012	**0.922**	**0.910**	0.012
$C_2$	ESN	0.897	0.897	**0.000**	0.625	**0.625**	**0.000**	0.985	0.985	0.000	0.737	**0.737**	**0.000**
$n_2=56$	RF	**0.946**	**0.939**	0.007	0.625	0.554	0.071	0.992	0.992	0.000	**0.753**	0.697	0.056
$C_3$	ESN	**0.929**	**0.912**	**0.017**	0.853	0.853	**0.000**	**0.985**	**0.985**	**0.000**	0.889	**0.881**	**0.008**
$n_3=61$	RF	0.889	0.803	0.086	**0.918**	**0.869**	0.049	0.973	0.950	0.023	**0.903**	0.835	0.068
$C_4$	ESN	**0.853**	**0.865**	**0.012**	**0.985**	**0.985**	**0.000**	**0.957**	**0.961**	**0.004**	**0.914**	**0.921**	**0.007**
$n_4=65$	RF	0.785	0.763	0.022	0.954	0.892	0.062	0.934	0.930	0.004	0.861	0.823	0.038
$C_\mathrm{bg}$	ESN	1.000	1.000	0.000	1.000	1.000	0.000	1.000	1.000	0.000	1.000	1.000	0.000
$n_\mathrm{bg}=69$	RF	1.000	1.000	0.000	1.000	1.000	0.000	1.000	1.000	0.000	1.000	1.000	0.000
w.m.	ESN	0.894	**0.893**	**0.001**	0.888	**0.888**	**0.000**	0.971	**0.971**	**0.000**	0.884	**0.884**	**0.000**
$n=321$	RF	**0.905**	0.876	0.029	**0.897**	0.866	0.031	**0.974**	0.966	0.008	**0.894**	0.860	0.034

The last row contains the weighted mean (w.m.) of the measures according to the class distribution in the test set. The differences between the performance at the default ($\varphi =0$) and the random starting angle (rnd. $\varphi _0$) are computed as absolute differences $\vert \Delta \vert$. The ESN recognized the same cells under different rotation angles more constantly. Superior results are printed in bold. The best hyper-parameters of the classifiers were chosen according to the best cross-validation results of experiment 2 (ESN: $N=4000$, RF: *forest size* $\, = 128$, *maximum tree depth* $\, = 12$)

The precision–recall curves and confusion matrices were quite diverse for the foreground classes. While curves for $C_3$ and $C_4$ were close to optimal, curves for other classes showed that the network had troubles discriminating among $C_1$ and $C_2$. This could also be observed by inspecting the confusion matrices in Fig. 9a, where the ESN frequently predicted $C_1$ when the true class was $C_2$. On the other hand, the RF was better in recognizing $C_1$ and $C_2$, but showed a tendency to predict $C_4$ when the true class was $C_2$ or $C_3$, and $C_3$, when the true class was $C_2$ or $C_4$, cf. Fig. 9b. The area under the curve (AUC) over all classes was also more stable for the RF. The control class $C_\mathrm{bg}$ has always been perfectly classified by both ESN and RF.

## Discussion and conclusions

While our previous work [24] focused on binary classification of similar cells from raw image patches, this paper showed that the proposed approach robustly generalizes to multi-class problems as well. Further, performance comparable to that of a random forest classifier that was trained the conventional way to achieve rotation-invariance, i.e., multiple rotated versions of an image patch, was observed. The model evaluation revealed that learning the temporal features works better for showing all rotations ($\Delta \varphi =0$) than for skipping some rotations ($\Delta \varphi >0$) when ESNs of the same complexity (in terms of reservoir capacity) were used. Yet, the ESNs were able to extract discriminative features in the generative mode, but significantly larger reservoirs were required to achieve similar classification performance. Future work could focus on evaluating the maximum period in generative mode with respect to the network’s test error.

We have considered short periods of zero-input between subsequent rotations of the same image to explicitly examine the capability of the reservoir to generate meaningful features from just a few external stimuli at specific points in a temporal input stream (experiments 2–4). However, this may not be an optimal setting for the ESN. Results from our model evaluations, especially with $\Delta \varphi =0$ in experiment 1, suggest that applying continuous input, i.e., without interruptions of the stream, may potentially deliver higher performance for this cell recognition task. That could for instance be realized by providing nonzero input only, such as keeping the input between consecutive rotations constant. Future work must additionally evaluate a more economical way of using the proposed rotation-invariant approach, such as subsampling the input sequence at specific rotations, which would additionally decrease the required runtime. However, any change to the ESN input scheme likely requires an adaption of hyper-parameters and may even lead to different architectures.

Considering the current approach, the runtime for feature computation in the reservoir could potentially be reduced by resetting the reservoir state instead of waiting until the resting state has been reached after the last rotation of an image has been presented. A first obvious advantage would be that we could use much shorter random periods between the inputs, or omit them entirely. This further would enable parallel training, since we could collect the temporal features from parallel copies of the reservoir and concatenate them (with, or without random gaps) before learning the regression weights. Similar holds for testing, where inference could be done for multiple images in parallel. However, it needs to be investigated critically, whether omitting the sequences of random length between individual images is feasible, and how this (positively or negatively) influences the performance on this task.

Interestingly, we could only observe a minor improvement in the baseline RF classification results when more training data were used. Even when we skipped 10 rotations, the mean overall accuracy was approximately as high as when we trained on all 360 rotations. From that we can conclude that the performance of the RF can only be slightly improved when we train on ten times the original dataset size. We presume that due to interpolation the images within a range of 10 subsequent rotation angles are too similar to increase the diversity in the training dataset and contribute to higher performance.

We see advantages in using ESNs for cell recognition: multiple classes can be learned from a single, randomly connected RNN, which is driven by raw image data. When compared to other, gradient descent-based training methods [47, 54], training via ridge regression is guaranteed to result in a global optimum. Besides the proposed regression inference scheme, more sophisticated (also nonlinear) schemes may lead to superior performance. Nevertheless, obtaining good hyper-parameters is highly task-specific and remains a tedious duty. Deep learning models, especially convolutional neural networks (CNNs [29]) have demonstrated good performance in white blood cell classification [17] and are therefore considered as a promising candidate in future research regarding the classification of subsequent maturation stages in the bone marrow. While CNNs also operate on raw images, they may be more robust in capturing the high intra-class variance while coping with small inter-class distance of blood cell maturation by learning significant features directly from the cell images. Nevertheless, robustly training deep feed-forward networks usually requires huge databases that are usually not available for biomedical imaging problems. Our approach, on the other hand, works well even with a small number of samples, and skewed class distributions [24].

We consider a classifier as *truly* rotation-invariant, if the same object can be recognized as the true class under arbitrary rotations. However, a rectangular image is bounded by definition, and when it gets rotated while keeping the original image dimensions, some parts of the rotated image may be undefined. A common strategy to overcome this problem is to use border extension techniques [15], for instance filling these regions with uniform gray values, or mirroring the border pixels. Depending on the ratio of the image dimensions, this may introduce significant mirroring artifacts and artificial repetitive patterns that do not contribute to the semantics of the image. Since we used square patches and the cell nuclei were centered, the introduced border artifacts are just minimal. Moreover, the proposed rotation scheme for the ESN training just considers pixels within the incircle of the square patch. Therefore, the border artifacts become negligible for both ESN and RF classifier. Nevertheless, the information in the original and rotated image is not completely equivalent, and therefore, we can only speak of achieving an *approximate* rotation-invariance. It has to be noted that despite the classifiers may have misclassified some samples in either the default (i.e., $\Delta \varphi _0=0$) or the random starting angle evaluation (see definition of experiment 4), the other one always resulted in a true positive recognition, cf. Fig. 10. Hence, to increase the overall recognition rates and make a step toward more robust rotation-invariant classifiers, it could be beneficial to classify a given test image several times under varying (e.g., random) angles and predict the final class label using some consolidation process.

The classification performance of the ESN on the presented bone marrow dataset has to be interpreted carefully, though. Firstly, due to the minimal inter-class distance that is caused by continuous maturation stages (i.e., $C_1$–$C_3$), and the cells’ appearance is frequently very similar and exacerbates finding a good (linearly) separating hyper-plane. Secondly, even after several years of experience, it is a non-trivial task for expert pathologists to make an exact distinction between consecutive maturation stages. The class distribution in this dataset might also slightly distort the results presented as weighted mean here, since $C_2$ is the minority—but most difficult—class to be recognized. This under-representation provokes a more optimistic view on our results, since the probability of misclassifying $C_2$ is lower due to the sample size. However, we provided per-class performance measures in our experimental results (Fig. 7) that showed non-consecutive maturation stages (i.e., $C_3$ and $C_4$) being recognized reliably even by less complex ESN models. Using a background class as control enabled assessing the discriminative power of the classifiers with respect to the foreground classes. However, in comparison to the foreground classes, the background class always shows very high classification accuracy in both classifiers. This behavior increases the overall mean accuracy measures and must be considered when interpreting the results. However, the collection of hundreds of images for each cell class requires the time-consuming, manual annotation by hematopathology experts. By employing label-preserving data augmentation strategies that mimic morphological variability we were able to generate more samples for this study. Our current research is focused on creating a larger bone marrow dataset to assess the robustness and generalization capability of the ESN and omit artificial data augmentation. Additionally, a thorough evaluation on other similar datasets is required to evaluate the transferability of the approach. Despite our promising results, an evaluation on a more extensive and fully balanced dataset obtained from multiple patients is required to derive more precise conclusions.

Previous work employed heterogeneous ensembles of classifiers [37, 41], where each individual instance focused on different aspects of the feature space, or even different features. They reported superior results of their ensembles over individual classifiers. Our results in experiment 4 revealed, that the RF classifier has weaknesses, where the ESN actually shows strengths—and vice versa, cf. Fig. 9. A combination of the two evaluated classifiers, i.e., ESN and RF, to increase the overall recognition rates in our experimental settings seems feasible. Deeper trees are expected to further increase the performance, but finding an optimum depth requires further examination. Using multiple ESNs in ensembles could be another opportunity to increase the performance by introducing diversity. One could use different settings for the individual networks, such as the sparsity of input and reservoir weights, different reservoir sizes, input weight scaling, and neuron models. Furthermore, training on different levels of a Gaussian scale space pyramid could add robustness against scale variations to a certain extent, where the linear regression would still guarantee globally optimal learning.

Using ESNs to classify bone marrow cells is attractive for applications in biomedical diagnostics due to the reliability of the system. An important measure in most medical application settings besides recall is a high specificity, as it is expressed by our recognition system. Since it is a learning-based strategy, it can more easily be transferred to other problems than rigid standard image processing approaches. A big advantage of the proposed approach is that cell segmentation and explicit manual feature extraction is not required, once the locations of cell nuclei are determined. The focus of this paper was set on discriminating blood cell maturation stages in the bone marrow, and thus we omitted including an automatic procedure to localize cell candidates in the histopathology images. Some previously reported approaches treated cell localization as a subproblem of cell counting, but we believe that a separation of matters regarding cell localization and cell classification has more potential. Recent work [26] presented state-of-the-art results in bone marrow cell localization with the ability to tune the detector toward producing a huge set of candidate cells. Such a reliable and accurate cell nuclei detection strategy could easily be employed as a preceding step before applying the rotation-invariant classification scheme proposed in this paper. Integrating these approaches into a fully automated system to support quantitative bone marrow diagnostics seems feasible and is subject of our future research.

## References

[1] Al-Janabi S, Huisman A, Van Diest PJ (2011). Digital pathology: current status and future perspectives. Histopathology.

[2] Bain BJ, Clark DM, Wilkins BS (2009). The normal bone marrow.

[3] Ballarò B, Florena AM, Franco V, Tegolo D, Tripodo C, Valenti C (2008). An automated image analysis methodology for classifying megakaryocytes in chronic myeloproliferative disorders. Med Image Anal.

[4] Bertsimas D, Tsitsiklis J (1993). Simulated annealing. Stat Sci.

[5] Bikhet S, Darwish A, Tolba H, Shaheen S (2000) Segmentation and classification of white blood cells. In: Proceedings of the 2000 IEEE international conference on acoustics, speech, and signal processing, 2000. ICASSP ’00, vol 6, pp 2259–2261. doi:10.1109/ICASSP.2000.859289

[6] Bishop CM (2006). Pattern recognition and machine learning.

[7] Breiman L (2001). Random forests. Mach Learn.

[8] Breiman L, Friedman J, Olshen R, Stone C (1984). Classification and regression trees.

[9] Cortes C, Vapnik V (1995). Support-vector networks. Mach Learn.

[10] Criminisi A, Shotton J (2013). Decision forests for computer vision and medical image analysis.

[11] Criminisi A, Shotton J, Konukoglu E (2012). Decision forests: a unified framework for classification, regression, density estimation, manifold learning and semi-supervised learning. Found Trends Comput Graphics Vis.

[12] Escalante HJ, Montes-y-Gómez M, González JA, Gómez-Gil P, Robles LA, García CAR, Reta C, Rosales-Pérez A (2012). Acute leukemia classification by ensemble particle swarm model selection. Artif Intell Med.

[13] Gençtav A, Aksoy S, Önder S (2012). Unsupervised segmentation and classification of cervical cell images. Pattern Recognit.

[14] Giuly RJ, Martone ME, Ellisman MH (2012). Method: automatic segmentation of mitochondria utilizing patch classification, contour pair classification, and automatically seeded level sets. BMC Bioinform.

[15] Gonzalez RC, Woods RE (2008). Digital image processing.

[16] Gurcan MN, Boucheron LE, Can A, Madabhushi A, Rajpoot NM, Yener B (2009). Histopathological image analysis: a review. IEEE Rev Biomed Eng.

[17] Habibzadeh M, Krzyzak A, Fevens T (2013) White blood cell differential counts using convolutional neural networks for low resolution images. In: Rutkowski L, Korytkowski M, Scherer R, Tadeusiewicz R, Zadeh LA, Zurada JM (eds) Artificial intelligence and soft computing. Springer, Berlin, pp 263–274. doi:10.1007/978-3-642-38610-7_25

[18] Hastie T, Tibshirani R, Friedman J (2013). The elements of statistical learning.

[19] Haykin S (1999). Neural networks—a comprehensive foundation.

[20] Hengen H, Spoor SL, Pandit MC (2002). Analysis of blood and bone marrow smears using digital image processing techniques. Proc SPIE.

[21] Jaeger H (2001) The “echo state” approach to analysing and training recurrent neural networks—with an erratum note. GMD Report 148, German National Research Center for Information Technology. http://www.faculty.jacobs-university.de/hjaeger/pubs/EchoStatesTechRep.pdf

[22] Jain A, Duin R, Mao J (2000). Statistical pattern recognition: a review. IEEE Trans Pattern Anal Mach Intell.

[23] Kainz P, Burgsteiner H, Asslaber M, Ahammer H, Iliadis L, Jayne C (2015). Robust bone marrow cell discrimination by rotation-invariant training of multi-class echo state networks. Engineering applications of neural networks—EANN 2015.

[24] Kainz P, Mayrhofer-Reinhartshuber M, Burgsteiner H, Asslaber M, Ahammer H (2014). Echo state networks for granulopoietic cell recognition in histopathological images of human bone marrow. Biomed Tech.

[25] Kainz P, Mayrhofer-Reinhartshuber M, Burgsteiner H, Asslaber M, Ahammer H (2014) The influence of image denoising on granulopoietic cell recognition using echo state networks. In: International biophysics congress. Brisbane

[26] Kainz P, Urschler M, Schulter S, Wohlhart P, Lepetit V (2015) You should use regression to detect cells. In: Navab N, Hornegger J, Wells WM, Frangi AF (eds) Medical image computing and computer-assisted intervention—MICCAI 2015 (Lecture notes in computer science), vol 9351, Springer International Publishing, pp 276–283. doi:10.1007/978-3-319-24574-4_33

[27] Khashman A (2008). IBCIS: intelligent blood cell identification system. Prog Nat Sci.

[28] Kim CH (2010). Homeostatic and pathogenic extramedullary hematopoiesis. J Blood Med.

[29] LeCun Y, Bottou L, Bengio Y, Haffner P (1998). Gradient-based learning applied to document recognition. Proc IEEE.

[30] Lempitsky V, Verhoek M, Noble JA, Blake A (2009) Random forest classification for automatic delineation of myocardium in real-time 3D echocardiography. In: Ayache N, Delingette H, Sermesant M (eds) Functional imaging and modeling of the heart: 5th international conference, FIMH 2009, Nice, France, June 3–5, 2009. Proceedings, Springer, Berlin, pp 447–456. doi:10.1007/978-3-642-01932-6_48

[31] Lin W, Xiao J, Micheli-Tzanakou E (1998) A computational intelligence system for cell classification. In: Proceedings of the 1998 IEEE international conference on information technology applications in biomedicine, pp 105–109. doi:10.1109/ITAB.1998.674687

[32] Lukoševicius M (2012) A practical guide to applying echo state networks. In: Montavon G, Orr GB, Müller KR (eds) Neural networks: tricks of the trade. Springer, Berlin, pp 659–686. doi:10.1007/978-3-642-35289-8_36

[33] Lukoševičius M, Jaeger H (2009). Reservoir computing approaches to recurrent neural network training. Comput Sci Rev.

[34] Maass W, Natschläger T, Markram H (2002). Real-time computing without stable states: a new framework for neural computation based on perturbations. Neural Comput.

[35] Markiewicz T, Osowski S, Marianska B, Moszczynski L (2005). Automatic recognition of the blood cells of myelogenous leukemia using SVM. Int Jt Conf Neural Netw IJCNN.

[36] Micheli-Tzanakou E, Sheikh H, Zhu B (1997). Neural networks and blood cell identification. J Med Syst.

[37] Mohapatra S, Patra D, Satpathy S (2014). An ensemble classifier system for early diagnosis of acute lymphoblastic leukemia in blood microscopic images. Neural Comput Appl.

[38] Ongun G, Halici U, Leblebicioglu K, Atalay V, Beksac M, Beksac S (2001). Feature extraction and classification of blood cells for an automated differential blood count system. Int Jt Conf Neural Netw IJCNN.

[39] Ramesh N, Dangott B, Salama ME, Tasdizen T (2012). Isolation and two-step classification of normal white blood cells in peripheral blood smears. J Pathol Inform.

[40] Ramoser H, Laurain V, Bischof H, Ecker R (2005) Leukocyte segmentation and classification in blood-smear images. In: IEEE-EMBS 2005. 27th annual international conference of the engineering in medicine and biology society, 2005, pp 3371–3374. doi:10.1109/IEMBS.2005.1617200 10.1109/IEMBS.2005.1617200 17280945

[41] Reta C, Altamirano L, Gonzalez JA, Diaz-Hernandez R, Peregrina H, Olmos I, Alonso Je, Lobato R (2015). Segmentation and classification of bone marrow cells images using contextual information for medical diagnosis of acute leukemias. PLoS One.

[42] Sabino DMU, Costa LF, Rizzatti EG, Zago MA (2004) Toward leukocyte recognition using morphometry, texture and color. In: IEEE international symposium on biomedical imaging: nano to macro, pp 121–124. doi:10.1109/ISBI.2004.1398489

[43] Scotti F (2005) Automatic morphological analysis for acute leukemia identification in peripheral blood microscope images. In: 2005 IEEE international conference on computational intelligence for measurement systems and applications CIMSA, pp 96–101. doi:10.1109/CIMSA.2005.1522835

[44] Shitong W, Min W (2006). A new detection algorithm (nda) based on fuzzy cellular neural networks for white blood cell detection. IEEE Trans Inf Technol Biomed.

[45] Sjöström PJ, Frydel BR, Wahlberg LU (1999). Artificial neural network-aided image analysis system for cell counting. Cytometry.

[46] Staroszczyk T, Osowski S, Markiewicz T (2012) Comparative analysis of feature selection methods for blood cell recognition in leukemia. In: Perner P (ed) Machine learning and data mining in pattern recognition. Springer, Berlin, Heidelberg, pp 467–481. doi:10.1007/978-3-642-31537-4_37

[47] Steil JJ (2007). Online reservoir adaptation by intrinsic plasticity for backpropagation–decorrelation and echo state learning. Neural Netw.

[48] Tai WL, Hu RM, Hsiao H, Chen RM, Tsai J (2011) Blood cell image classification based on hierarchical SVM. In: IEEE international symposium on multimedia (ISM), pp 129–136. doi:10.1109/ISM.2011.29

[49] Theera-Umpon N (2005) White blood cell segmentation and classification in microscopic bone marrow images. In: Wang L, Jin Y (eds) Fuzzy systems and knowledge discovery (Lecture notes in computer science), vol 3614, Springer, Berlin, pp 787–796. doi:10.1007/11540007_98

[50] Theera-Umpon N, Dhompongsa S (2007). Morphological granulometric features of nucleus in automatic bone marrow white blood cell classification. IEEE Trans Inf Technol Biomed.

[51] Theera-Umpon N, Gader P (2000) Training neural networks to count white blood cells via a minimum counting error objective function. In: Proceedings of the 15th international conference on pattern recognition, vol 2, pp 299–302. doi:10.1109/ICPR.2000.906072

[52] Venkatalakshmi B, Thilagavathi K (2013) Automatic red blood cell counting using hough transform. In: 2013 IEEE conference on information communication technologies (ICT), pp 267–271. doi:10.1109/CICT.2013.6558103

[53] Verstraeten D, Dambre J, Dutoit X, Schrauwen B (2010). Memory versus non-linearity in reservoirs. Int Jt Conf Neural Netw IJCNN.

[54] Werbos PJ (1990). Backpropagation through time: what it does and how to do it. Proc IEEE.

[55] Woodward A, Ikegami T (2011) A reservoir computing approach to image classification using coupled echo state and back-propagation neural networks. In: International conference image and vision computing, Auckland, New Zealand, pp 543–458

[56] Wu Q, Zeng L, Ke H, Xie W, Zheng H, Zhang Y (2005). Analysis of blood and bone marrow smears using multispectral imaging analysis techniques. Proc SPIE.

[57] Xie Z, Gillies DF (2016). Patch forest: a hybrid framework of random forest and patch-based segmentation. Proc SPIE.

[58] Zheng Q, Milthorpe BK, Jones AS (2004). Direct neural network application for automated cell recognition. Cytom Part A.

[59] Zheng X, Zhang Y, Shi J, Yu Y (2011) Analysis of leukemia development based on marrow cell images. In: 2011 4th international congress on image and signal processing (CISP), vol 1, pp 95–99. doi:10.1109/CISP.2011.6099937

[60] Zheng X, Zhang Y, Shi J, Yu Y (2011) A new method for automatic counting of marrow cells. In: 2011 4th international conference on biomedical engineering and informatics (BMEI), vol 1, pp 42–46. doi:10.1109/BMEI.2011.6098263

